# Neurosurgical Anesthesia: Optimizing Outcomes with Agent Selection

**DOI:** 10.3390/biomedicines11020372

**Published:** 2023-01-27

**Authors:** Andrew Nguyen, Akhil Mandavalli, Michael Joseph Diaz, Kevin Thomas Root, Aashay Patel, Jed Casauay, Priyanka Perisetla, Brandon Lucke-Wold

**Affiliations:** 1 College of Medicine, University of Florida, Gainesville, FL 32611, USA; 2 Department of Neurosurgery, University of Florida, Gainesville, FL 32608, USA

**Keywords:** agent selection, neuroanesthesia, neurological surgery, total intravenous anesthesia, volatile anesthesia

## Abstract

Anesthesia in neurosurgery embodies a vital element in the development of neurosurgical intervention. This undisputed interest has offered surgeons and anesthesiologists an array of anesthetic selections to utilize, though with this allowance comes the equally essential requirement of implementing a maximally appropriate agent. To date, there remains a lack of consensus and official guidance on optimizing anesthetic choice based on operating priorities including hemodynamic parameters (e.g., CPP, ICP, MAP) in addition to the route of procedure and pathology. In this review, the authors detail the development of neuroanesthesia, summarize the advantages and drawbacks of various anesthetic classes and agents, while lastly cohesively organizing the current literature of randomized trials on neuroanesthesia across various procedures.

## 1. Development and Adoption of Anesthesia in Neurosurgery

The role of anesthetics in neurosurgical interventions is one that has had a storied history. In antiquity, crude, homeopathic anesthetic interventions were thought to accompany trephination procedures and early craniotomies, and found moderate advancements across the Paleolithic age to the Renaissance [1]. The implementation of anesthesia for surgical interventions in the form of chloroform, ether, and nitrous oxide were developed throughout the early to mid-19th century, following the work of pioneers such as William Morton, Sir Humphrey Davy, and Dr. Crawford W. Long [1]. However, the formation of neuroanesthesiology as a field would follow advancements in neurosurgical interventions in the following decades; beginning in the late-1800s to early 1900s, surgeons such as Dr. Victor Horsley, Dr. Fedor Krause, Dr. Emil Theodor Kocher, and Dr. Harvey Cushing would utilize anesthesia in the forms of chloroform, ether, and ethyl chloride for various animal studies and neurosurgeries, and local anesthetic interventions utilizing derivatives of cocaine would also find footing [1]. By the mid-1900s, the study of anesthetic implementations in neurosurgery began flourishing under the likes of Dr. Albert Faulconer and Dr. John D. Michenfelder, and the first formal textbook in neuroanesthesia was written by Dr. Andrew Hunter in 1964 [2]. Following the advancements in the field from the early to mid-20th century, developments in neuroanesthesia have been steady as research directions now focus on understanding the mechanisms of brain injury and neuroprotection, physiological and pharmacological effects of anesthetic agents on relevant neuroanatomy, and effective practice through induction, maintenance, and limitations of the use of neuroanesthesia clinically [2].

General anesthetic agents can be categorized as those administered intravenously or inhaled, the latter of which can be further subdivided into either volatile or non-volatile agents [3]. As the methods of administration vary, so do the proposed mechanisms of action for these substances; intravenous agents such as propofol, etomidate, and barbiturates facilitate the uptake of chloride anions and subsequently improve the inhibitory response of pentameric GABAa receptors of the thalamus and the reticular activating system. Such actions promote unconsciousness, establishing the commonality of their use in the induction phase of anesthesia [4]. Non-volatile inhalation agents such as nitrous oxide, xenon and cyclopropane are used heavily in the maintenance of anesthesia, as their inhibitory effects on NMDA receptors and 2-Pore-domain K^+^ channels help reduce downstream postsynaptic depolarization events and ultimately improve analgesia and sedation [5]. The intravenous agent ketamine has been proposed to act upon this receptor as well [6]. Volatile agents that are inhalable, such as halothane, flurane, isoflurane, sevoflurane, and desflurane act on various receptors within the central nervous system to foster analgesia, immobility, and loss of consciousness [7]. These receptors not only include the aforementioned GABAa receptors, 2-pore-Domain-K^+^ channels, and NMDA receptors but also serotonin receptors, neuronal nicotinic acetylcholine receptors, sodium channels, and potassium channels [4,8].

## 2. Physiological Effects of Anesthesia during Neurosurgery 

As neuroanesthetic induction and maintenance must balance the preservation of stable hemodynamics and perfusion of cerebral tissues with alterations to intracranial pressure that such perfusion may incur, it is pertinent to note the systemic effects of these various anesthetic agents in neurosurgical procedures [3]. This consideration not only ensures that the metabolic demands of the organs of the central nervous system are met, but also reduces injury and trauma to them. Most anesthetic agents, with the exception of ketamine, reduce the overall global oxygen metabolism of the brain, whereas the cerebral blood flow is increased by volatile anesthetics, nitrous oxide, and ketamine but reduced by other intravenous agents [3]. Such changes in physiological parameters impinge upon several endogenous mechanisms including cerebral autoregulation, vasomotor reactivity, and neurovascular coupling, of which anesthetic agents have varied effects [3].

Cerebral autoregulation entails adjustments to vascular tone that are sensitive to both long-term and short-term changes in systemic blood pressure [9]. These changes are hypothesized to be regulated myogenically or endothelially in response to stress on the vasculature, neurogenically by the autonomic nervous system and neurons, and to some extent metabolically [10]. Volatile anesthetic agents have been found to reduce cerebral autoregulation at higher doses, and propofol in particular has been found to reduce it at levels >200 mcg/kg/min [3]. In addition, synthetic opioids have been found to increase cerebral blood flow by vasodilation as a result of such cerebrovascular autoregulation [11]. The vasomotor reactivity of cerebral arterioles arises from pH changes to vasculature and surrounding smooth muscles, and as such, is largely dependent on the partial pressure of carbon dioxide: increases in PaCO_2_ have been shown to have vasodilatory effects, increasing blood flow [3]. The vasomotor reactivity is reduced at higher concentrations of certain volatile agents and has been shown to be variably affected by propofol [3]. Neurovascular coupling can be thought of as a combination of phasic and tonic vasomodulation regulated by the production of substances and metabolites by neurons and astrocytes, respectively. The role of these various anesthetic agents on neurovascular coupling has yet to be fully determined but they have been shown to affect neural activity as well as subsequent vasoactive signal transmission, and vasoactive response as well [3,12]. Most notably, etomidate, propofol, and barbiturates have been found to decrease cerebral blood flow secondary to decreased cerebral metabolic demands [3].

Intracranial pressure, determined by the pressure within the skull exerted by its content in the form of blood volume, tissue volume, and cerebrospinal fluid volume, is functionally related to cerebral perfusion pressure, and affected by overall cerebral blood flow [13]. As such, alterations in cerebral blood flow following the use of anesthetic agents subsequently coincide with changes in intracranial pressure, as demonstrated through the Cushing reflex equation:

(1) CPP = MAP − ICP

where CPP is cerebral perfusion pressure, MAP is mean arterial pressure, and ICP is intracranial pressure [10].

As Bazin (1997) notes that as intravenous agents decrease cerebral blood flow secondary to their effects on cerebral metabolic rates, there are subsequent decreases in intracranial pressure [11]. Intracranial pressure appears to increase proportionally to increases in cerebral blood flow induced by various volatile inhalation agents up to a certain concentration, as well as intravenous ketamine, leading to their cautionary use in the induction and maintenance of anesthesia [11]. Non-volatile inhaled agents have mixed effects on cerebral autoregulatory mechanisms, and as such, have varied effects on cerebral blood flow and intracranial pressure [3]. Nitrous oxide has historically been found to increase cerebral blood flow and increased intracranial pressure, especially within patients with intracranial lesions, whereas Xenon has been found to decrease cerebral blood flow and reduce intracranial pressure [3,14]. As synthetic opioids increase cerebral blood flow following autoregulatory vasodilation as a response to systemic hypertension, such anesthetic agents have been found to increase intracranial pressure as well [11].

## 3. Overview of Classes of Neuroanesthesia

### 3.1. Total Intravenous Anesthetics 

The first mention of total intravenous anesthesia (TIVA) in the literature dates to 1872 in a report by Pierre-Cyprine describing the use of chloride hydrate [15]. Following the introduction of propofol in 1977, the archetypal TIVA, TIVA utilization rapidly increased [16]. Propofol increases GABA-mediated chloride channels in the brain resulting in inhibitory tone in the CNS. Furthermore, the drug increases the duration of the GABA’s effects by decreasing dissociation from its receptor, leading to the hyperpolarization of the cell membrane [17]. Its effects have a rapid onset and are short-acting, which allows for rapid recovery post-surgery and evaluation of neurological function [18]. Propofol also decreases intracranial pressure, cerebral blood flow, cerebral metabolism, and edema while supporting cerebral perfusion pressure and mean arterial pressure. This aggregate of effects is neuroprotective during cerebral ischemia [19]. Furthermore, the administration of propofol with an opioid provides hypnosis, amnesia, and minimizes response and movement to surgical stimulation, achieving all of the factors of a true anesthetic while also decreasing postoperative nausea and vomiting [16,18]. TIVA is now generally classified as the combined use of a hypnotic agent (e.g., propofol) and an opioid (e.g., fentanyl or remifentanil) without concurrent use of inhaled anesthetics [20,21]. Additionally, TIVA is conducive to new considerations that have emerged in neurosurgery anesthesia such as minimizing affected brain function and electrophysiological monitoring. Due to propofol’s rapid onset and short duration, infusion rates can be adjusted to allow for patient cooperation when necessary during a procedure [18]. Current common intravenous agents include thiopentone, propofol, etomidate, ketamine, benzodiazepines, and opioids [19].

### 3.2. Volatile Anesthetics 

Diethyl ether was identified centuries ago and may have been originally compounded by an 8th-century Arabian philosopher. However, it was not until 1842 that the first use of ether for surgical anesthesia was documented [22]. More than 150 years after this discovery of a form of general anesthesia, volatile agents are still in clinical use today [23]. While the precise mechanism of action of volatile anesthetics remains largely unknown, studies have demonstrated that they are active in the central nervous system, augmenting GABA receptors and stimulating potassium channels [8]. Additionally, these agents have a role in depressing excitatory pathways including acetylcholine receptors, glutamate receptors, serotonin receptors, muscarinic receptors, and nicotinic receptors [8]. They are most often administered through a face mask, laryngeal mask, or endotracheal tube. While induction with volatile agents is often preferred in infants and young children because it allows for a needleless experience while awake, adult patients typically undergo an intravenous induction to reduce risks inherent to a full-grown patient. In a randomized double-blind comparison of 8% sevoflurane and propofol as anesthetic agents, Thwaites et al. found that induction with sevoflurane was associated with a lower rate of apnea, decreased time to establish spontaneous ventilation, and a smoother transition to maintenance. Furthermore, emergence was found to be earlier with sevoflurane [24]. While volatile agents have few absolute contraindications beyond gene variants for malignant hyperthermia, in the field of neurosurgery, these agents have concerns as they have been shown to decrease cerebral perfusion and increase intracerebral pressure [21,25]. Some of the most common volatile anesthetics in use today include halothane, isoflurane, desflurane, and sevoflurane.

### 3.3. Advantages and Disadvantages of Anesthetic Options

When administering anesthesia to neurosurgical patients, the onus is on the anesthetist to gauge whether inhaled or intravenous anesthetics would be more advantageous. Three of the most significant considerations regarding a neurosurgical anesthetic are the effects on hemodynamics, intracranial pressure, and postoperative conditions [19]. Hemodynamic stability is needed to maintain cerebral autoregulation [26]. Furthermore, the intrinsic autoregulation of factors (e.g., partial CO_2_ pressure and mean arterial pressure) that influence hemodynamic stability may be lost via the circumstances precipitating neurosurgical management [27,28]. Therefore, hemodynamic consideration is especially important within the context of neurosurgical anesthesia. In a randomized study, Strebel et al. demonstrated that volatile agents can impair autoregulation while propofol is helpful in preserving it [29]. Furthermore, Van Hemelrijck et al. demonstrated that the use of a propofol-loading infusion did not change the measure of blood pressure or heart rate, demonstrating the benefits of TIVA within the context of hemodynamics [30]. Low intracranial pressure is a significant factor allowing for optimal operating conditions and can also be neuroprotective. In a randomized prospective study, Petersen et al. found that subdural intracranial pressure was lower and cerebral perfusion pressure was higher in patients anesthetized by propofol compared to sevoflurane- or isoflurane-anesthetized patients [31]. This finding suggests that TIVA is beneficial in neurological surgery by minimizing local hypoperfusion and cerebral ischemia. Following surgery, rapid recovery from anesthesia is important to allow for neurological examination. Regarding the duration of effect, both TIVA with propofol-remifentanil and volatile agents have short half-lives, allowing for a rapid recovery after surgery [32]. This is supported in the literature as demonstrated by a randomized sample of patients undergoing craniotomy. Here, Talke et al. demonstrate that there was no difference between early post-operative recovery variables such as recovery time and early cognition [33]. Despite the benefits of TIVA demonstrated here, the ideal choice of anesthetics for neurosurgery remains controversial [34]. An appraisal of the present literature of randomized-controlled trials comparing anesthetic agents head-to-head across various peri and postoperative parameters are included in Table 1 and Table 2 [30,31,32,33,35,36,37,38,39,40,41,42,43,44,45,46,47,48,49,50,51,52,53,54,55,56,57,58,59,60,61,62,63,64,65,66,67,68,69,70,71,72]. The authors detail the search process below.

### 3.4. Systematic Review of Randomized-Controlled Trials Comparing Neuroanesthetic Agents (Spine or Spinal or Vertebrae or Vertebra) and (Brain or Cranial or Cranium) and (Surgery or Operation or Operative) and (Anaesthesia or Anesthesia or Sedative) and (ICP or Intracranial Pressure)

Pubmed, Web of Science, and Scopus databases were queried with the depicted search criteria. Rayyan Web App for Systematic Reviews [73] was utilized for article selection. The initial query revealed 6259 articles after removal of duplicates. Abstract and title screening excluded 5003 articles that were not RCTs. Full-text and article review produced 42 articles of which all were kept for final inclusion in Table 1 and Table 2. The selection process was developed and conducted by two authors (A.N. and A.M.). Inclusion criteria included (1) randomized-controlled trials, (2) directly comparing general anesthetic agents (i.e., not opioids, analgesics) head-to-head, and (3) the use of neurological surgeries (either cranial or spinal). Exclusion criteria were (1) not randomized-controlled trials, (2) involving surgeries not related to cranial or spinal operations, (3) non-English, and (4) non-human studies.

## 4. Biological Mechanism of Widespread, Select TIVA Agents

### 4.1. Thiopental

Prior to its discontinuation in 2011, thiopental existed as the prevalent TIVA in operative neurosurgery. [74] In its final decade, newer general anesthetic agents including etomidate, ketamine, dexmedetomidine, and propofol had gradually solidified their presence in the operating room, partially contributing to the decision to cease thiopental production. The clinical equipoise surrounding these three latter agents concerns their effects on important surgical parameters including ICP, CPP, CMRO_2_, and emergence time, as previously discussed, among other factors. 

### 4.2. Propofol 

The anesthetic ability of propofol is attributed to its affinity to a specific subset of gamma-aminobutyric acid (GABA) receptors—GABA_A_ receptors [75]. The GABA_A_ receptors are ligand-gated ion channels that facilitate the influx of chloride ions into the neurons of the CNS. The consequent influx creates a state of hyperpolarization and inhibition, precisely targeted by many modern anesthetic agents, including propofol [76,77,78,79]. Propofol binds in the region between the beta and alpha subunits of GABA_A_R, intensifying the inhibitive polarization induced by neurotransmitter GABA and GABA_A_R binding. Clinically, propofol has been demonstrated to decrease ICP, CMRO_2_ emergence time (particularly important in neurosurgery which hinges on early assessment of postoperative CNS status), while maintaining CPP [63,80]. Its drawbacks, often regarding secondary variables, include its administrative dependence with analgesic agents for co-injection—its introduction has been found to create ineligible pain without any independent analgesic ability [81]. Similarly, to propofol, the mechanisms of etomidate and thiopental are attributed to their agonistic effects on GABA_A_ receptors which ultimately increase the postsynaptic inhibition on projection neurons [82]. Likewise, etomidate and thiopental have demonstrated their capacity to decrease ICP and cerebral metabolic rate of oxygen (CMRO_2_) and maintain a steady CPP [83,84,85]. Contrastingly, these two agents lack the rapid half-life propofol possesses, gearing particular interest toward propofol when mitigating delays in postoperative assessment and operative costs. 

### 4.3. Ketamine

**Figure 1:**
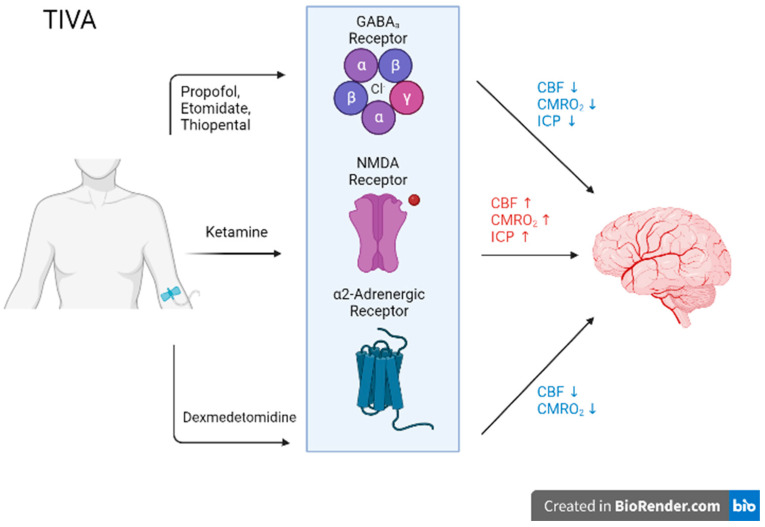
The proposed mechanism of action for total intravenous anesthetic agent (TIVA), including receptor type affected and subsequent effects on cerebral blood flow (CBF), cerebral metabolic rate of oxygen (CMRO2), as well as intracranial pressure (ICP). Differing TIVA agents act on different receptors, for which varying effects on cerebral blood flow and oxygen consumption are a byproduct. Created with Biorender.com.

Ketamine bivalently binds to the NMDA receptor as a non-competitive antagonist, preventing the excitatory effects of glutamate binding. This leads to a reduction in calcium influx, synaptic transmission, and polarization [83,84,85,86]. Despite its analgesic efficacy, it has been observed to display considerable drawbacks, often associated with ICP elevation and CPP [87]. However, few systematic reviews have suggested ketamine to adequately maintain ICP and CPP, and in certain instances lower ICP [88]. Dexmedetomidine acts as an agonist on a family of G-protein-coupled receptors, alpha-2 adrenoreceptors in the CNS, particularly in the locus ceruleus [82,89]. These receptors display inhibitory effects, specifically targeting adenylyl cyclase activity, repressing it, initiating a downstream cascade, ultimately inducing hyperpolarization. Regarding its effect on clinical parameters, dexmedetomidine may impose a risk of CPP deterioration [90,91]. The literature surrounding its ability to reduce ICP is ambiguous with further trials and cohort studies required to establish a reliable stance on this aspect. The action of these agents is illustrated in Figure 1. 

## 5. Biological Mechanism of Widespread, Select Inhaled Agents 

### 5.1. Sevoflurane

Sevoflurane’s anesthetic effect suggests that it enhances the inhibitory postsynaptic channel activity of GABA and glycine while inhibiting the excitatory synaptic channel activity of NMDA, nicotinic acetylcholine, serotonin, and glutamate in the CNS [92]. It is a halogenated anesthetic that is delivered by vaporizer to the lungs [92]. The low blood/gas coefficient of sevoflurane (conferring titratability) plus the following properties proffer favorability in the neurosurgical setting: rapid onset, rapid offset, and nondistinctive disturbance of cerebral hemodynamics [93]. Indeed, in long-duration neurosurgical cases (mean minimum alveolar concentration (MAC) hours: 4.7), intracranial surgery patients anesthetized with sevoflurane (40% O_2_) reported shorter time to emergence and postoperative neurological assessment than did patients anesthetized with isoflurane (40% O_2_) (*n* = 60) [94]. Other independent randomized controlled trials have since corroborated this basic result, supporting sevoflurane to achieve faster emergence, particularly in pediatric neurosurgical populations [52,95]. A retrospective study of preconditioning with isoflurane, sevoflurane, or desflurane also supported sevoflurane usage for its relative lack of airway irritation and smoother emergency under clinical conditions [96]. Still, however, concerns surrounding sevoflurane’s neurotoxic potential remain, owing to its high rate of metabolism and reaction with carbon dioxide absorbents [93]. Zhou and colleagues concluded that sevoflurane–remifentanil inhalation correlated with a higher incidence of intraoperative hypotension, brain edema, and post-operative nausea and vomiting, compared to intravenous propofol–remifentanil [97].

### 5.2. Desflurane 

Desflurane appears to affect the lipid bilayer of the neuronal membrane, disrupting neural synaptic transmission. These agents may also work by blocking the excitatory ion channels and increasing the activity of the inhibitory ion channels [98]. It is an alternative halogenated ether characterized by high saturated vapor pressure, minimal metabolism, and short duration of action [98]. Similar to sevoflurane, desflurane confers markedly improved awakening properties over gold standard isoflurane, and both have near-identical impacts on cerebral blood flow [52,99]. Magni et al. reported that craniotomy patients receiving desflurane with end-tidal of 6–7% (1.2 MAC) had a shorter extubation and recovery time than did craniotomy patients anesthetized with sevoflurane with end-tidal of 1.5–2% (1.2 MAC) (*n* = 120). [32] Indeed, at the same MAC, desflurane has also been evidenced to have a stronger inhibitory effect than sevoflurane [100]. There too exists data suggesting that desflurane administration may alter the neuro. Hoffman et al. reported that 9% end-tidal desflurane improved brain tissue metabolic status following craniotomy if mean arterial pressure is maintained [101].

### 5.3. Isoflurane

**Figure 2:**
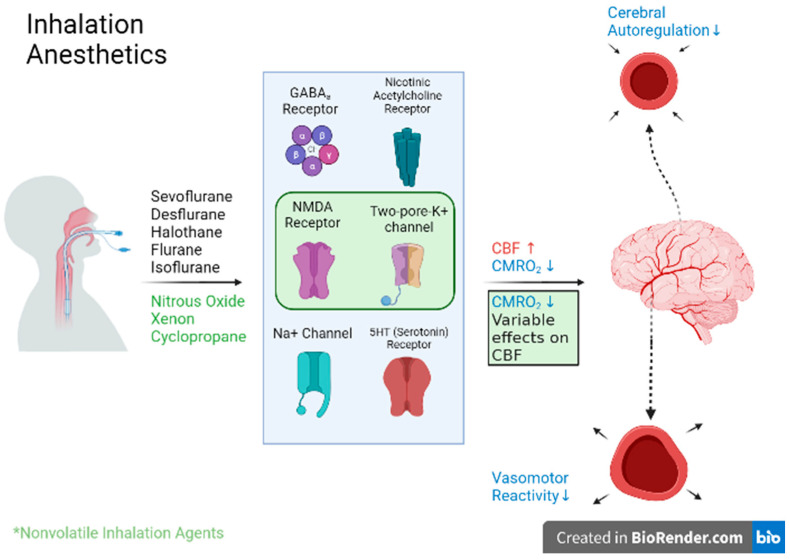
The proposed mechanism for inhalation anesthetics, including both nonvolatile (highlighted in green) and volatile anesthetics. Effects on CBF and CMRO2 are largely dependent on whether the agent is volatile or nonvolatile, but changes depicted to vasomotor reactivity and cerebral autoregulation have largely been studied in volatile anesthetics alone. Created with Biorender.com.

Isoflurane binds to GABA, glycine, and *N*-methyl-D-aspartate (NMDA) receptors in the CNS to inhibit the activity of neurotransmitter-gated ion channels, which may promote skeletal muscle relaxation [102]. It is considered the choice inhalational agent for neuroanesthesia [93]. In sham-controlled trials, 2% isoflurane was sufficient for a significant reduction in brain injury and inflammation post-subarachnoid hemorrhage, potentially via the sphingosine kinase axis [103,104]. However, isoflurane has also been demonstrated to mediate neurotoxicity (namely via RohA activation and mitochondrial dysregulation), which leads to downstream endoplasmic reticulum-associated stress [105,106], In the event of isoflurane neurotoxicity, Cheng et al. found that the melatonergic agonist agomelatine may be a useful additive for reduction in inflammation and damage induced by isoflurane neurotoxicity [107]. Noted increases in lumbar cerebrospinal fluid pressure have also been a cause for concern in normocapnic neurosurgical patients receiving inhaled anesthesia [37,40]. Though Adams and colleagues previously established that hypocapnia is not required prior to isoflurane induction to avoid increases in cerebrospinal fluid pressure increases, late results contraindicate isoflurane administration in normocapnia in patients with neuropathologies. [108,109,110] Taken together, these findings support the neuroprotective effect of isoflurane following intracranial hemorrhage and elucidate clear methods for safe and efficacious counteraction. The action of these agents is illustrated in Figure 2. 

## 6. Neuroanesthesia in Cranial Operations

### 6.1. Craniotomies 

Although there exists a wide variety of anesthetic agents, electing which agents to use can vary greatly depending on the surgical procedure being performed. In awake craniotomy, the typical agents of choice for local anesthetic are bupivacaine mixed with lidocaine and epinephrine, with the primary intent of local anesthetic administration avoiding intravenous opioid administration [111]. However, no consensus on choice or dose of local anesthetic for awake craniotomy currently exists. In a systematic review and meta-analysis of anesthesia management for awake craniotomy, Stevanovic et al. found that agents for choice for regional selective scalp nerve block, a local anesthesia delivery method, ranged from bupivacaine only, ropivacaine only, or mixtures of either bupivacaine, ropivacaine, and/or epinephrine [112]. Local anesthetic choices for RSNBs vary due to complications such as local anesthetic toxicity and hypertension, with the latter especially relevant in the case of inadvertent intravascular epinephrine injection [111,113].

As for the preferred choice of sedative and general anesthetic agents for awake craniotomy, primary choices include propofol, remifentanil, and dexmedetomidine. Propofol, in comparison, with dexmedetomidine, exhibited a lower incidence of intraoperative seizures, but a longer arousal time from asleep phase to awake phase [114,115]. Remifentanil is often found supplemented with propofol, and has the advantage of being easily titratable and less prone to causing gastrointestinal complications than fentanyl [111]. Dexmedetomidine is also often used in conjunction with propofol and/or remifentanil, and has been shown to lead to shorter arousal times and higher surgeon satisfaction intra- and post-operatively [111]. Rapid arousal post-operatively is beneficial in order to ensure proper electrocortical mapping, and thus this property of dexmedetomidine displays a clear advantage for using this particular agent [116,117].

### 6.2. Functional Interventions

For electroconvulsive therapy (ECT), a commonly preferred anesthetic agent is propofol, for its various improvements in efficacy and less resulting complications compared to other anesthetic agents. Propofol was found to be linked to shorter seizure duration and lower increases in heart rate and blood pressure intra-operatively as found in a review by Rasmussen, with these shorter propofol-related seizures and lower changes in heart rate and blood pressure (as compared to thiamylal, another anesthetic agent) also supported in a study on propofol’s effect on cognitive recovery by Sakamoto et al. [118]. Although the difference in resulting functional outcomes such as improvement of depressive symptoms and cognitive ability recovery were found to be minor when comparing patients treated with propofol and methohexital, Geretsegger et al. found propofol to be preferable over methohexital due to its shorter seizure duration and lower increase in blood pressure, a similar finding to studies comparing propofol to thiamylal [119]. 

The recent use of ketamine for ECT anesthesia has also sparked an interest for a further investigation of its efficacy. In a case series of 14 patients who received ketamine in place of methohexital, all 14 patients reported dissatisfaction with the drug due to elicitation of nausea, vomiting, dizziness, and in some cases, dissociative phenomena [116]. Despite this, ketamine can be advantageous due to its rapid action and antidepressive effects, and can be paired with other anesthetic agents to offset some of its negative side effects, such as increased cardiac excitation [120]. In a study of 48 patients divided into three groups of 16, the efficacy of propofol only, ketamine only, and propofol and ketamine use for ECT were studied; although the propofol and ketamine group experienced higher seizure intensity and duration, they also resulted in faster and larger improvements in their depressive symptoms, as quantified by the Hamilton Depression Rating Scale [120]. These studies point to a possible efficacious use of ketamine as an anesthetic agent for ECT, as long as potential negative side effects are properly managed.

## 7. Neuroanesthesia in Spinal Operations

### 7.1. Spinal Cord Injuries

It is of the utmost importance that the anesthetic agents selected for induction during neurosurgical procedures provide optimal patient management without potentially exacerbating life-threatening symptoms. Among the cases that require such cautious management are treatments of spinal cord injuries (SCIs) [121]. SCIs refer to a broad category of insults to the spinal cord at any vertebral level that can transiently or permanently alter its function and can occur through an acute or chronic disease process [122]. For acute SCIs, surgical intervention involving spinal decompression and stabilization is often a crucial aspect of preventing spinal compression from producing local spinal cord ischemia, thereby avoiding a potential secondary injury [122]. However, particularly at the cervical and higher thoracic levels, SCI frequently disrupts sympathetic pathways regulating blood pressure adaptability and can cause persistent hypotension, a significant complicating factor that necessitates special consideration when selecting an induction agent [123].

Currently, there does not exist a specific guideline that requires any particular inductive anesthetic for decompressive surgery treating SCI, and additionally, there is no existing research that has demonstrated superior patient outcomes of decompressive spinal surgery with any particular anesthetic agent [121]. Despite this, the deliberate avoidance of anesthetic-induced hypotension and choice of intraoperative neurophysiological monitoring for the purpose of observing spinal cord function deterioration narrow down preferable anesthetic options. For example, the use of certain TIVA anesthetic agents such as propofol may cause severe hypotension in hypovolemic SCI patients [121,124]. In this case, it is recommended that these agents are simultaneously balanced with ketamine, which increases axial pressure, to avoid endangering the patient [121,125].

Furthermore, special consideration for anesthetic agents must be conducted when a SCI decompressive surgery necessitates the use of intraoperative neurophysiological monitoring to preserve neural functionality. Somatosensory-evoked potentials (SSEPs) are the most prevalent measure of intraoperative neurophysiological monitoring for decompressive surgeries, but are subject to evoked potential depression by volatile anesthetics, such as isoflurane, and prolonged latency in measurements with sevoflurane [121,126,127,128,129]. Therefore, in surgeries utilizing SSEP monitoring, it is recommended to either use TIVA exclusively or balance TIVA with low doses of volatile anesthetics [121]. Additionally, motor-evoked potentials (MEPs) and muscle motor-evoked potentials are commonly used to monitor the motor pathways, but due to their movement-inducing nature, it is more advantageous to use TIVA agents instead of volatile anesthetics [121,130,131]. Furthermore, nondepolarizing neuromuscular blocking agents, such as rocuronium, cisatracurium, and vecuronium can be considered when recording MEPs but must be closely monitored by the team anesthesiologist [132,133]. Alternatively, spontaneous electromyography can be utilized to monitor nerve root function from peripheral musculature recordings in real-time [121,126]. Inhaled anesthetics are preferred because spontaneous electromyography cannot be conducted with the administration of neuromuscular blockades [121,134].

### 7.2. Elective Procedures

Although pathologies related to elective spinal procedures are less acute in nature compared to that of spinal cord injuries, the complexity of their corrective surgeries and the consideration of the anesthetic agent require equal attention. Among the most common reasons for patients to undergo elective spinal procedures is lumbar spinal stenosis (LSS), a condition characterized by pain associated in the lower extremities and buttocks, sometimes concurrent with lower back pain, that is caused by a narrowing of the vertebral canal and/or intervertebral foramina where spinal nerve elements transmit [135,136]. After conservative, nonsurgical alternative treatments fail to alleviate symptoms, surgeries such as laminectomies are electively considered to achieve spinal decompression [135,137]. Another common spinal condition that can be electively corrected via surgery is adjacent segment disease (ASD). ASD is a broad spectrum of complications that arise from spinal fusion or laminectomy, possibly due to the additional biomechanical stress that is exerted on adjacent vertebral bones as a result of decreased spinal movement [136,138]. ASD is preferred to be treated as conservatively and minimally invasive as possible, but should these options fail, an additional spinal fusion is warranted [139,140].

There are two primary choices of anesthetic modalities available with lumbar laminectomies and spinal fusions: general anesthesia and neuraxial anesthesia. General anesthesia is typically selected for patients where intraoperative airway management is deemed to be challenging [141]. For the selection of anesthetic agents, the considerations made for elective surgeries are similar to those of SCI decompressive surgeries, including cases with intraoperative neurophysiological monitoring. Neuraxial anesthesia is normally in the form of spinal anesthesia for lumbar laminectomies, which entails injecting anesthetic agent locally into the cerebrospinal fluid (CSF) to achieve anesthetic effects on exiting lumbosacral nerves [142]. Anesthetic agents used for spinal anesthesia include ropivacaine and bupivacaine [143]. A major contraindication for using spinal anesthesia with bupivacaine involves inducing hypotension in patients, usually due to hypovolemia [144]. Analyses between general anesthesia and spinal anesthesia demonstrate that both modalities are equally safe and effective for lumbar laminectomies, although patients with spinal anesthesia may have reduced nausea and postoperative complications and require a shorter period of anesthesia [145,146]. Despite the current lack of documentation of spinal anesthesia in spinal fusions, recent studies have determined similar potential benefits as those seen with use in lumbar laminectomies [147,148]. 

General anesthesia is most commonly used in elective spine surgeries such as microdiscectomy and lumbar decompression procedures. In patients undergoing spinal laminectomy, dexmedetomidine-based total intravenous anesthesia is suitable and provides stable perioperative hemodynamic response. Turgut et al. observed that dexmedetomidine-based infusion compared to fentanyl-based required less post-operative analgesics (*p* < 0.01) and resulted in less frequent post-operative nausea and vomiting (*p* < 0.01) [70]. Dexmedetomidine has also been demonstrated to reduce opioid requirements in the intraoperative period [149]. During lumbar spinal surgery, controlled hypotension may be used to reduce blood loss; Albertin et al. found that blood loss and intraoperative bleeding were significantly reduced when propofol was used compared with sevoflurane (*p* < 0.05) due to differences in selective vasodilation [69]. The quality of emergence from anesthesia is an important consideration in cervical spine surgery for postoperative neck stabilization and neurological assessment. In a study by Inoue et al., a fentanyl-based regimen in cervical spine surgery patients resulted in less bucking (*p* < 0.05) and perception of pain (*p* < 0.01) and a shorter time to extubation (*p* < 0.01) than sevoflurane only [67].

Regional neuraxial anesthesia has been demonstrated to have several short-term advantages over general anesthesia, including reduced intraoperative hypertension and tachycardia, postoperative nausea and vomiting, and postoperative analgesic requirement [150]. It may be preferred for patients undergoing simple lumbar surgeries with no contraindications to neuraxial analgesia (such as coagulopathy, infection at site of needle, hypovolemia, and spinal abnormalities) [150]. Isobaric bupivacaine 0.5% has been shown to be suitable and is indicated over hyperbaric bupivacaine for higher levels of sensory block and fewer hemodynamic events [151]. Additionally, an enhanced recovery after major surgery (ERAS) protocol using a modified thoracolumbar interfascial plane (mTLIP) block can reduce opioid requirements in both laminectomies (*p* < 0.03) and spinal fusion procedures (*p* < 0.04) [152].

## 8. Conclusions

Anesthesia in neurosurgery embodies a vital element in the development of neurosurgical intervention. This undisputed interest has offered surgeons and anesthesiologists an array of anesthetic selections to utilize, though with this allowance comes the equally essential requirement of implementing a maximally appropriate agent. Broadly separated into two categories, neuroanesthesia can be viewed as either intravenous or inhaled, both pairs granting unique advantages. In this review, we highlight the various benefits and shortcomings of such agents and coalesce the current body of randomized controlled trials of general anesthetic agents across procedures such as craniotomies for hemorrhage, resection of neoplasms, deep brain stimulation, among others in addition to spinal surgeries such as for deformity correction or emergent trauma.

## Figures and Tables

**Table 1:** Published trials of general anesthetic agents for cranial procedures.

Authors and Year	Surgical Procedure	Comparison	Findings
Aken et al., 1990 [35]	Unspecified cranial procedure	Balanced anesthesia (loading thiopental and fentanyl + maintenance fentanyl, droperidol, thiopental, and isoflurane in nitrous oxide, *n* = 20) vs. TIVA (loading propofol + alfentanil infusion, *n* = 20)	During induction, TIVA had a significantly greater hemodynamic stability. Balance anesthesia was associated with a significantly longer emergence time than TIVA.
Hemelrijck et al., 1991 [30]	Craniotomy for resection of brain tumor	Propofol (*n* = 20) vs. thiopental (*n* = 20)	Postoperative return to orientation time was shorter in the propofol group (7 +/− 5 min vs. 27 +/− 23 min).
Ornstein et al., 1993 [36]	Craniotomy for resection of supratentorial lesion	Anesthetic maintenance via desflurane (*n* = 12) vs. isoflurane (*n* = 12)	CBF values were non-significantly different as measured at 1 MAC and 1.5 MAC concentrations for both desflurane and isoflurane (*p* > 0.05), as well as at 1.25 MAC as measured in *n* = 15 patients (*p* > 0.05).
Talke et al., 1996 [37]	Hypophysectomy	Propofol (*n* = 10) vs. loading propofol + maintenance desflurane (*n* = 10) vs. loading propofol + maintenance isoflurane (*n* = 10)	Minimum CPP was significantly lower in desflurane (*p* < 0.05) and isoflurane (*p* < 0.05) groups compared to propofol-only control. Minimum SBP was significantly lower in desflurane (*p* < 0.05) and isoflurane (*p* < 0.05) compared to propofol-only control.
Artru et al., 1997 [38]	Unspecified cranial procedure	Anesthetic maintenance via sevoflurane (*n* = 8) and isoflurane (*n* = 6) following induction via mannitol	Neither sevoflurane or isoflurane significantly altered ICP, and both decreased middle cerebral artery flow velocity (Vmca). Notably, decreased Vmca with sevoflurane was related to decreased CPP at 0.5 MAC (*p* < 0.05), and increased CVRe at 1.0 and 1.5 MAC (*p* < 0.05). The CPP decreased from baseline at 0.5, 1.0, and 1.5 MACs of isoflurane (*p* < 0.05).
Hoffman et al., 1998 [39]	Craniotomy for unspecified pathology	Thiopental induction (*n* = 10) vs. desflurane (*n* = 10)	Neither thiopental nor desflurane changed tissue gases or pH, but desflurane increased PO_2_ 70% (*p* < 0.05), whereas thiopental decreased PO_2_ 30% during temporary brain artery occlusion.
Talke et al., 1999 [40]	Transsphenoidal Hypophysectomy	Anesthetic maintenance via propofol (*n* = 10) vs. sevoflurane (*n* = 20)	Sevoflurane increased lumbar CSF pressure and decreased CPP and systolic blood pressure following infusion while propofol did not affect lumbar CSF pressure, CPP, nor systolic blood pressure.
Talke et al., 2002 [33]	Craniotomy for resection of supratentorial lesion	Propofol (*n* = 20) vs. isoflurane (*n* = 20)	Emergence time to eyes opening was not different between anesthetic agents (*p* > 0.05). There was no difference in occurrence of hypertension (*p* > 0.05).
Iwata et al., 2003 [41]	Unspecified intracranial surgery	Propofol (*n* = 13) vs. sevoflurane (*n* = 13)	There was no difference in the rate of temperature decrease and recovery in induced hypothermia (*p* < 0.05).
Fraga et al., 2003 [42]	Craniotomy for resection of supratentorial lesion	Inhalation of isoflurane (*n* = 30) vs. desflurane (*n* = 30) following induction via fentanyl, thiopental, and vecuronium maintained with 60% nitrous oxide in oxygen	There were no significant differences between MAP, ICP, and CPP between use of desflurane and isoflurane, but notable decreases (*p* < 0.05) in both groups from baseline values with regard to MAP and CPP. The ratio between the cerebral metabolic oxygen requirement and cerebral blood flow decreased significantly for both groups as well.
Petersen et al., 2003 [31]	Craniotomy for resection of supratentorial tumor	Propofol (*n* = 41) vs. isoflurane (*n* = 38) vs. sevoflurane (*n* = 38)	No differences in ICP or CPP between anesthetic agents (*p* > 0.05).
Günes et al., 2005 [43]	Unspecified intracranial procedure	Anesthetic maintenance via propofol (*n* = 39) vs. dexmedetomidine (*n* = 39)	Systolic blood pressure and MAP were not different between the two agents. Extubation time was shorter for propofol (*p* < 0.05). Analgesic requirements were higher for propofol (*p* = 0.013).
Magni et al., 2005 [32]	Supratentorial craniotomy for unspecified pathology	Propofol (*n* = 64) vs. sevoflurane (*n* = 64)	Emergence time was not different between anesthetic agents. Occurrence of hypertension was higher in propofol than sevoflurane use (*p* = 0.0046), and hypotension was higher in propofol than sevoflurane (*p* = 0.02).
Sekimoto et al., 2006 [44]	Craniotomy for resection of brain tumor	Anesthetic maintenance via halothane vs. isoflurane vs. sevoflurane after induction via propofol/fentanyl/nitrous oxide	Halothane, isoflurane, and sevoflurane were all found to reduce systolic blood pressure, but only sevoflurane and isoflurane decreased train-of-four ratios significantly at 1.0 MAC (*p* < 0.001). Amplitudes of transcranial motor-evoked potentials were reduced by isoflurane and sevoflurane at 0.5 MACs, but not halothane, reflecting the reduced extent of the neuromuscular blockade initiated by halothane.
Djian et al., 2006 [45]	Unspecified intracranial procedure	Remifentanil vs. sufentanil in combination with propofol for maintenance of anesthesia	Remifentanil was associated with the need for less adjustments with regard to hemodynamic stability (*p* = 0.037), greater use of morphine (*p* = 0.01), and higher intraoperative opioid costs. However, there was no significant differences in extubation times between groups.
Bhagat et al., 2008 [46]	Craniotomy for unspecified pathology	Anesthetic maintenance via propofol (*n* = 50) vs. isoflurane (*n* = 50)	Hypertension occurrence and MAP change were not different between the two agents. Emergence time was higher for propofol (*p* = 0.008).
Bonhomme et al., 2009 [47]	Unspecified intracranial procedure	Propofol (*n* = 30) vs. sevoflurane (*n* = 31)	Propofol was associated with higher occurrence of intraoperative hypertension (*p* < 0.001) and sevoflurane was associated with higher occurrence of intraoperative hypotension (*p* = 0.015).
Ali et al., 2009 [48]	Resection of pituitary tumor	Propofol (*n* = 30), isoflurane (*n* = 30), sevoflurane (*n* = 30)	Emergence time was significantly longer with use of isoflurane (*p* < 0.001). Hypertension occurrence was higher in isoflurane than in propofol or sevoflurane, and higher in sevoflurane than propofol (*p* < 0.001). Hypotension was not difference between anesthetics (*p* = 0.36).
Bilotta et al., 2009 [49]	Craniotomy for resection of supratentorial lesion	Sevoflurane (*n* = 28) vs. desflurane (*n* = 28)	Significant delays in cognitive “awakening” for obese and overweight patients receiving sevoflurane-based anesthesia as compared to those receiving desflurane-based anesthesia as measured by post-operative short orientation memory concentration test scores at 15 and 30 min (*p* < 0.005, *p* < 0.005) as well as with the Rancho Los Amigos scale (*p* < 0.005)
Güneş et al., 2009 [71]	Craniotomy for resection of supratentorial lesion	Anesthetic maintenance with dexmedetomidine in addition to sevoflurane (*n* = 30), desflurane (*n* = 30), and isoflurane (*n* = 30)	MAP was elevated following intubation for all groups. Rates of eyes opening and responsiveness following verbal commands were lower in desflurane–dexmedetomidine than in other groups (*p* = 0.001).
Magni et al., 2009 [72]	Craniotomy for resection of supratentorial lesion	Anesthetic maintenance via sevoflurane (*n* = 60) vs. desflurane (*n* = 60)	Mean emergence was similar between the two groups, but extubation and recovery time were lower (*p* < 0.001) in the desflurane group. Hemodynamic stability differences were non-significant between the two groups.
Lauta et al., 2010 [50]	Craniotomy for resection of supratentorial lesion	Anesthetic maintenance via propofol (*n* = 153) vs. sevoflurane (*n* = 149)	Propofol was associated with a significantly longer emergence time to eyes opening (*p* < 0.014. Sevoflurane was associated with higher occurrence of hypotension (*p* < 0.0167).
Yildiz et al., 2011 [51]	Craniotomy for resection of supratentorial lesion	Anesthetic maintenance via desflurane (*n* = 35) vs. isoflurane (*n* = 35)	Heart rate was not different between the two agents. MAP was higher for desflurane (*p* < 0.05). Extubation time and eyes opening time was shorter for desflurane (*p* < 0.05).
Ghoneim et al., 2015 [52]	Craniotomy for resection of supratentorial tumors	Anesthetic maintenance via isoflurane (*n* = 20) vs. sevoflurane (*n* = 20) vs. desflurane (*n* = 20)	Emergence times were significantly shorter for desflurane or sevoflurane than with isoflurane in pediatric patients following a craniotomy for supratentorial tumors.
Hernandez et al., 2015 [53]	Craniotomy for hematoma	Anesthetic maintenance via propofol (*n* = 20) or sevoflurane (*n* = 20)	SSEPs amplitudes and latencies were not different between the two agents. TceMEPs amplitudes were higher for propofol (*p* < 0.05). Latencies were shorter in the propofol group (*p* < 0.05).
Goettel et al., 2016 [54]	Awake craniotomy for unspecified pathology	Dexmedetomidine (*n* = 25) vs. propofol (*n* = 25)	There were no differences in level of sedation (OAA) (*p* = 0.13). There were no differences in intraoperative hypertension (*p* = 0.60), hypotension (*p* = 0.50), or complications (*p* = 0.99). There was no difference in postoperative complications (*p* > 0.05).
Gokcek et al., 2016 [55]	Unspecified intracranial procedure	Anesthetic maintenance via sevoflurane (*n* = 25) vs. desflurane (*n* = 25)	Emergence time and time to eyes opening were higher with sevoflurane (*p* < 0.001).
Lin et al., 2016 [56]	Resection of supratentorial lesion	Anesthetic maintenance via propofol (*n* = 31) vs. dexmedetomidine (*n* = 31)	NIHSS-positive change was higher in propofol than dexmedetomidine (*p* < 0.001). Focal neurologic deficits were higher in propofol than dexmedetomidine (*p* < 0.05).
Rajan et al., 2016 [57]	Craniotomy or transsphenoidal approach for resection of brain tumor	Dexmedetomidine (*n* = 68) vs. remifentanil (*n* = 71)	Dexmedetomidine was associated with significantly lower postoperative MAP (*p* < 0.001). Dexmedetomidine was associated with significantly longer emergence time to open eyes (*p* < 0.001).
Thongrong et al., 2017 [58]	Craniotomy for unspecified pathology	Anesthetic maintenance via fentanyl (*n* = 30) vs. dexmedetomidine (*n* = 30) after propofol induction	Dexmedetomidine infusions reduced adverse effects, with signs of effectively controlled systolic blood pressure one minute prior to skull pin insertion (*p* < 0.05), as well as during skull pin insertion (*p* < 0.01) in comparison to fentanyl. Similarly, dexmedetomidine infusions were related to reduced adverse hypertensive and hypotensive responses in patients.
Bhardwaj et al., 2018 [59]	Surgical clipping for aneurysmal subarachnoid hemorrhage	Propofol (*n* = 35) vs. desflurane (*n* = 35)	There was no difference in blood loss (*p* < 0.05), hypotension (*p* < 0.05), hypertension (*p* < 0.05), or emergence time for eyes opening (*p* < 0.05).
Gracia et al., 2018 [60]	Unspecified intracranial procedure	Anesthetic induction via propofol (*n* = 20) vs. thiopental (*n* = 20)	There was no difference in heart rate (*p* > 0.05). MAP was significantly higher in thiopental groups (*p* < 0.05). Systolic and diastolic blood pressure was significantly lower in thiopental groups (*p* < 0.05).
Molina et al., 2018 [61]	Craniotomy for resection of tumor	Propofol–remifentanil (*n* = 105) for asleep sedation vs. conscious sedation with dexmedetomidine (*n* = 75)	Patients sedated with dexmedetomidine used less opiates, antihypertensive drugs, and had a lower postoperative duration and length of stay (all *p* < 0.001).
Xinyan et al., 2018 [62]	Awake craniotomy for unspecified pathology	Dexmedetomidine (*n* = 20), propofol (*n* = 20), etomidate (*n* = 20)	There was no significant difference in perioperative wake up duration (*p* > 0.05) and postoperative emergence time (*p* > 0.05). The rate of adverse events was lower in dexmedetomidine than propofol and etomidate (*p* < 0.05). The rate of adverse events was lower in propofol than etomidate (*p* < 0.05).
Khallaf et al., 2019 [63]	Craniotomy for hematoma	Anesthetic maintenance via propofol (*n* = 20) vs. dexmedetomidine (*n* = 20)	Tachycardia, bradycardia, and hypertension occurrences were not different between the two agents. IPP and CPP changes were not different between the two agents. Hypotension occurrences were higher in the propofol group (*p* = 0.024).
Preethi et al., 2021 [64]	Craniotomy for hematoma	Anesthetic maintenance via propofol (*n* = 45) vs. isoflurane	Change in heart rate, systolic blood pressure, diastolic blood pressure, and MAP were not different between the two agents. Brain relaxation was higher for propofol (*p* < 0.05). ICP was higher for isoflurane (*p* = 0.01).
Balasubramanian et al., 2021 [65]	Surgical clipping/endovascular coiling for aneurysmal subarachnoid hemorrhage	Propofol (*n* = 8) vs. isoflurane (*n* = 8) vs. sevoflurane (*n* = 8), vs. desflurane (*n* = 8)	There was no significant difference found between anesthetic on levels of CSF caspase-3 levels.

**Table 2:** Published trials of general anesthetic agents for spinal procedures.

Authors and Year	Surgical Procedure	Comparison	Findings
Laureau et al., 1999 [66]	Posterior instrumentation for treatment of idiopathic scoliosis	Induction via intravenous propofol (*n* = 15) vs. midazolam (*n* = 15)	Cortical somatosensory-evoked potentials did not deteriorate in either the propofol or the midazolam induction groups.
Inoue et al., 2005 [67]	Cervical spine surgery for unspecified pathology	Anesthetic maintenance via fentanyl and propofol (*n* = 25) vs. fentanyl and <1% sevoflurane (*n* = 25) vs. sevoflurane (*n* = 25)	Perception of pain and bucking scores following emergence- were greater for patients exposed to sevoflurane versus propofol and fentanyl and fentanyl and sevoflurane in combination.
Kurt et al., 2005 [68]	Unspecified spinal procedure	Anesthetic maintenance via isoflurane (*n* = 12) vs. sevoflurane (*n* = 10) vs. desflurane (*n* = 10)	Sevoflurane and isoflurane administered via volatile anesthetics were able to achieve controlled hypotension in comparison to desflurane with systolic blood pressures outside the target range of 32% and 26% for isoflurane and sevoflurane, respectively, and 44% with desflurane.
Albertin et al., 2008 [69]	Lumbar spine surgery for unspecified pathology	Induction via sevoflurane (*n* = 14) or propofol (*n* = 14) as main anesthetic agents	Peripheral blood flow was greater in the propofol group before and during the hypotensive period, but had reduced blood loss and intra-operative bleeding as compared to the sevoflurane group (*p* < 0.005).
Turgut et al., 2008 [70]	Lumbar laminectomy	Pre-operative bolus and anesthetic maintenance via dexmedetomidine (*n* = 25) vs. fentanyl (*n* = 25) following induction via propofol as well as maintenance	Extubation and discharge times were similar between dexmedetomidine and fentanyl, but MAP values after intubation for those exposed to dexmedetomidine were higher for those exposed to fentanyl before and after extubation. Supplemental analgesia was required earlier for fentanyl group patients (34.8 +/− 1.35 min vs. 60.4 +/− 1.04 min).

## Data Availability

Not applicable.

## Abbreviations

CPP—cerebral perfusion pressure; MAP—mean arterial pressure; ICP—intracranial pressure; TIVA—total intravenous anesthesia; CMRO_2_—cerebral metabolic rate of oxygen; SCI—spinal cord injury; SSEPs—somatosensory-evoked potentials; MEPs—motor-evoked potentials; LSS—lumbar spinal stenosis; ASD—adjacent segment disease; ERAS—enhanced recovery after major surgery; mTLIP—modified thoracolumbar interfascial plane.
